# Gα3 subunit Thga3 positively regulates conidiation, mycoparasitism, chitinase activity, and hydrophobicity of *Trichoderma harzianum*

**DOI:** 10.1186/s13568-020-01162-9

**Published:** 2020-12-17

**Authors:** Jie Ding, Jie Mei, Pei Huang, Ying Tian, Yao Liang, Xiliang Jiang, Mei Li

**Affiliations:** 1 Institute of Plant Protection, Chinese Academy of Agricultural Sciences, No. 2 West Yuanmingyuan Rd., Haidian District, Beijing, 100193 China; 2 College of Plant Protection, Yunnan Agricultural University, National Key Laboratory for Conservation and Utilization of Biological Resources in Yunnan, Kunming, 650201 China

**Keywords:** Thga3, *Trichoderma harzianum*, Conidiation, Mycoparasitism, Chitinase, Hydrophobicity

## Abstract

Heterotrimeric G-proteins are key elements of signal transduction pathways, which participate in regulating multiple biological processes in fungi including growth, conidiation, antagonism, and mycoparasitism. Among G protein subunits, Gα3 showed diverse regulatory functions in different fungi. In this study, we cloned a Gα3 subunit coding gene *thga3* from *T. harzianum* Th33 that can antagonize *Rhizoctonia solani* and some other plant pathogenic fungi. A *thga3* deletion strain Δ*thga3* was generated using the double-crossover homologous recombination strategy, and R*thga3* was generated by transforming *thga3*-expressing vector into the protoplasts of Δ*thga3* by the PEG/CaCl_2_-mediated method. The biological characteristics of wild-type Th33, Δ*thga3* and R*thga3* were evaluated. Compared with wild-type Th33, Δ*thga3* showed 15%, 94%, and 23% decrease in hyphal growth, conidia yield, and chitinase activity, respectively, and Δ*thga3* showed lower antagonistic and mycoparasitism abilities, while there were no significant differences between wild-type Th33 and R*thga3*. The hyphal surface hydrophobicity of Δ*thga3* significantly decreased compared with those of the wild-type Th33 and R*thga3*. qRT-PCR analysis revealed that transcript abundance of the hydrophobin gene (tha_09745) of Δ*thga3* decreased by 80% compared with that of wild-type Th33 and R*thga3*. The results showed that *thga3* positively regulates the growth, conidiation, hydrophobicity, chitinase activities, and mycoparasitism of Th33 towards *R. solani*. We hence deduced that the expression level of Tha_09745 is correlated to the hyphal hydrophobicity of Th33 and therefore affects the other biological characteristics of Th33. The findings of this report provide a foundation for elucidating the G-protein signal regulatory mechanisms of fungi.

## Introduction

*Trichoderma* spp. are widely used as biocontrol agents. Understanding their genetic regulation of biocontrol activities is beneficial to their genetic improvement and application as biofungicides. The biocontrol effects of *Trichoderma* preparations against plant pathogenic microorganisms are affected by a variety of factors, including development stage, environmental temperature, humidity, light, and nutrition. The current understanding of how *Trichoderma* senses external signals, transmits the external signals to the cells, and regulates growth and development is limited. G proteins play key roles in regulating the growth, development, reproduction, pathogenesis, and secondary metabolite biosynthesis in filamentous fungi (Nogueira et al. 2015; Lei et al. 2019). Heterotrimeric G-protein complexes consist of alpha (α), beta (β), and gamma (γ) subunits. Most filamentous fungi have three Gα subunits, namely, Gα1, Gα2, and Gα3. Different Gα subunits regulate different biological processes in fungi (Lei et al. 2019). Gα1 subunits are found more frequently and regulate vegetative growth, conidiation, and mycoparasitic responses in fungi (Rocha-Ramírez et al. 2002; Sun et al. 2016; Reithner et al. 2005). The function of Gα2 in fungi is rarely reported (Lei et al. 2019), and Gα3 subunits possess different regulatory functions, the understanding of which is based on studies on Gα3 subunits from *Trichoderma* spp. (Schmoll et al. 2009; Susanne et al. 2005), *Penicillium* spp*.* (García-Rico et al. 2017; Hu et al. 2013), *Valsa mali* (Song et al. 2017), and *Fusarium* spp. (Yu et al. 2008; Guo et al. 2016). These regulate the growth, conidiation, cellulase, and chitinase activities of fungi. Several Gα3 subunits from various *Trichoderma* strains exhibit relatively different functions. For example, GNA3 (Gα3) from *T. reesei* (do Nascimento et al. 2009) and GNA3 (Gα3) in *T. reesei* (Schmoll et al. 2009) are related to cellulase activity, while Tga3 (Gα3) in *T. viride* affects chitinase gene expression (Susanne et al. 2005) and Tga3 (Gα3) in *T. atroviride* and GNA3 (Gα3) in *T. viride* negatively regulate conidiation. Light seems to play a role in regulating functions of Gα3 subunits in *Trichoderma* (Schmoll et al. 2009; Susanne et al. 2005). Studying the functions of Ga3 subunits of different *Trichoderma* strains may further clarify their functions and regulatory mechanisms.

This study cloned a Th33 Gα3 gene, *thga3*, that can antagonize multiple plant pathogenic fungi, including *Rhizoctonia solani*, *Fusarium* spp. and *Phytophthora* spp. In addition, we observed that the function of this gene differs from that of previously reported Gα3. It positively regulates the growth, conidiation, and chitinase activities as well as the mycoparasitism ability of Th33 on *R. solani*. Both hyphal hydrophobicity and the expression of a type II hydrophobin gene Tha_09745 in Th33 are positively regulated by Thga3. These findings indicate that the hydrophobicity of Th33 is correlated to the expression of Tha_09745 and therefore influences the biological characteristics of Th33.

## Materials and methods

### Strains and culture conditions

The strains used in this study include wild-type *T. harzianum* Th33 (CGMCC No. 19906), mutant Δ*thga3* (*thga3* deletion strain), mutant R*thga3* (*thga3* complemented strain), and pathogenic fungi *R. solani* (ACCC No. 36124). For microscopic observation and mycelial biomass determination, strains were inoculated into potato dextrose agar (PDA) and incubated at 25 °C for 4 days. *Escherichia coli* Trans1-T1 competent cells were purchased from TransGen (TransGen Biotech, Beijing, China) and used for cloning and propagation of plasmids. Hygromycin B (hyg, 200 mg/mL) was added to PDA to screen Δ*thga3*, and geneticin (G418 sulfate, 100 mg/mL) was used for screening R*thga3*.

### Deletion of *thga3* in *T. harzianum* Th33

**Fig. 1:**
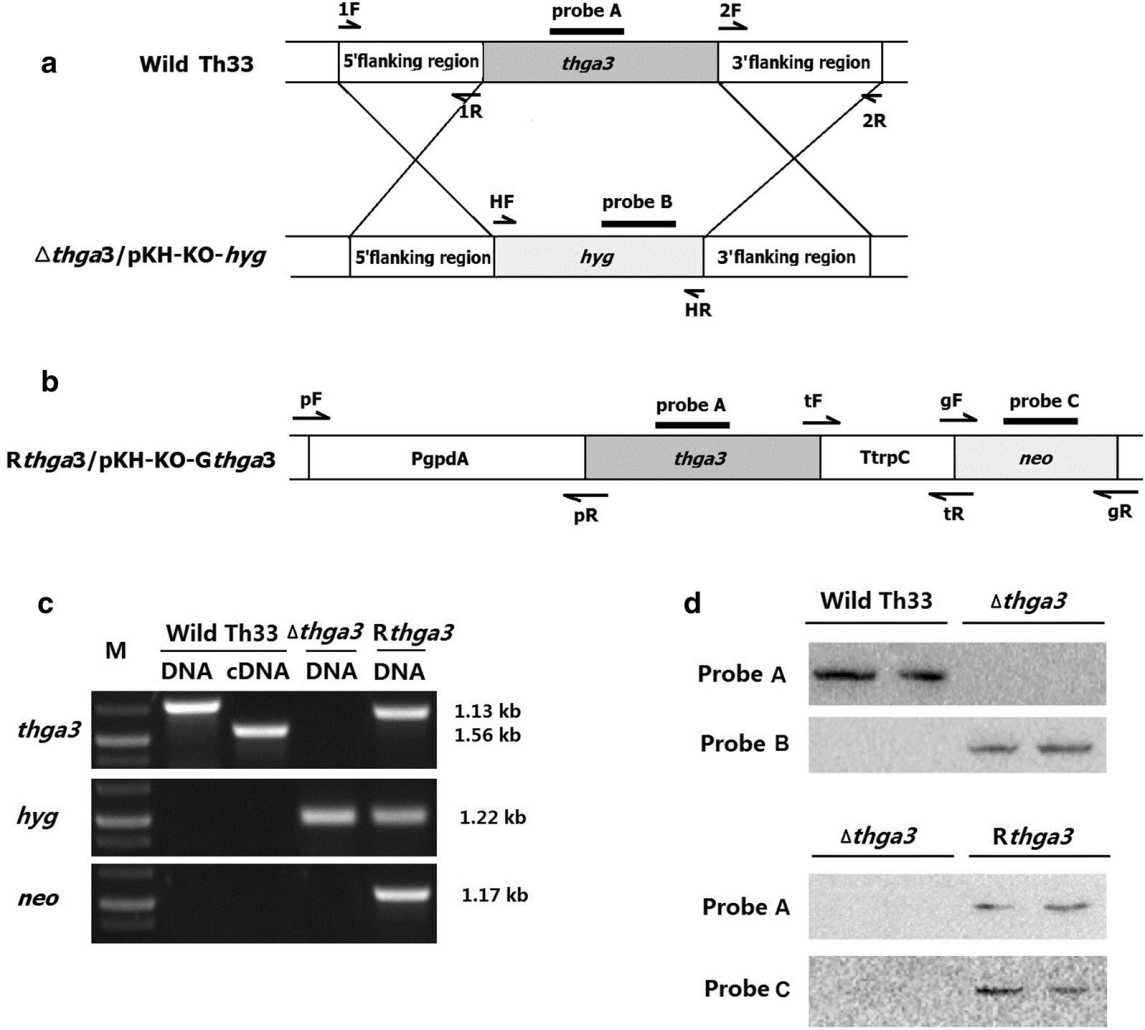
Construction of *thga3* deletion and complemented mutants. **a** *Thga3* gene deleting strategy. **b** *Thga3* gene complementing vector pKH-KO-G*thga3*. **c** PCR and RT-PCR identification of *thga3*, *hyg*, and *neo* genes in Δ*thga3* and R*thga3*. M, 250-bp ladder. PCR templates were genomic DNA and cDNA of wild-type Th33, genomic DNAs of Δ*thga3* and R*thga3*. **d** Southern hybridization analysis. Probe A. *Thga3* ORF; Probe B. *Hyg* gene; ProbeC. *Neo* gene. DNAs of wild-type Th33, Δ*thga3* and R*thga3* were digested by *EcoR*I/*Xho*I

The Gα3 gene *thga3* of *T. harzianum* Th33 was cloned from the Th33 genomic DNA (GenBank Accession Number PRJNA272949) (Sun et al. 2016). The *thga3* deletion strain Δ*thga3* was generated by the double-crossover homologous recombination strategy. The deletion strategy is shown in Fig. 1a using the primers listed in Additional file 1: Table S1. Plasmid pKH-KO (Wang et al. 2014) was used as a transformation vector containing two uracil-specific excision reagent (USER) cloning sites, USC1 and USC2, on either side of the hygromycin B gene *hyg* (Wang et al. 2014). The 5′ flanking region (1,110 bp) of the *thga3* coding sequence was cloned from genomic DNA of Th33 and cloned into the USC2 sites of HindIII/*Xho*I-digested pKH-KO using Clontech In-Fusion®HD Cloning Kit (TaKaRa). The 3′ flanking region (1036 bp) was cloned into the USC1 sites of SpeI/EcoRI-digested pKH-KO in the same orientation as that of the 5′ flanking region to generate the *Thga*3 disruption vector pKH-KO-*thga*3. The PEG/CaCl_2_-mediated method (Aragona and Valente, 2015) was used to generate the Δ*thga3* by transforming pKH-KO-*thga*3 into the protoplasts of wild-type Th33, and the genotypes of Δ*thga3* mutants were confirmed by amplifying internal fragments of *thga3* (no PCR product generated), and the *hyg* fragment (PCR product was 1221 bp in size).

### Complementation of *thga3*

To generate the complemented strain of the *thga3* deletion mutant, a *thga3* complementation cassette containing the promoter PgpdA, terminator TtrpC, and the coding region of *thga3* was constructed. PgpdA and TtrpC fragments were amplified from the plasmid pAN7-1. The TtrpC fragment was first fused to the genome of wild-type Th33 and ligated with *thga3* to produce chimeras using gene splicing by overlap extension (SOE) (Shevchuk et al. 2004). The derived chimeras were used as a template for amplifying the fragment containing *thga3* and TtrpC sequences, which was then fused with the PgpdA using SOE and generated the *thga3* complementation cassette, which was then cloned into *Spe*I/*EcoR*I-digested pKH-KO-G using a Clontech In-Fusion®HD cloning kit. The resulting complementing vector was designated as pKH-KO-G*thga3* (Fig. 1b). pKH-KO-G*thga3* was then transferred into Δ*thga3* protoplasts. The *thga3* complemented strain R*thga3* was screened by G418 resistance (100 mg/mL), and the genotypes were confirmed by PCR amplifying internal fragments of *thga3* and *neo* (G 418 resistance gene).

Reverse-transcription polymerase chain reaction (RT-PCR) was used to confirm the transcription of *thga3* in wild-type Th33 and the mutants. Total RNA was isolated using TransZol Up Plus RNA. cDNA was prepared from total RNA using FastQuant RT kit (With gDnase) (Tiangen). R-Taq polymerase (TaKaRa) was used for PCR amplification of *thga3* from cDNAs.

### Southern blotting

Southern hybridization was performed using a DIG-High prime DNA labeling and detection starter kit II (Roche, Germany) according to the manufacturer’s protocol. Fragments of *thga3* [Probe A, 371 bp], *hyg* [selective marker gene, probe B (426 bp)], and *neo* [selective marker gene, probe C] were amplified for use as probes. DNAs of wild-type Th33 and mutants Δ*thga3* and R*thga3* were digested by EcoRI/XhoI. The primers used in this assay are listed in Additional file 1: Table S1.

### Growth, conidiation, and hydrophobicity

Wild-type Th33, Δ*thga3*, and R*thga3* were respectively inoculated at the center of PDA plates and cultured for 5 days at 25 °C. The colony morphology of each strain was monitored, and radial hyphal growth rates were measured daily. Twenty culture discs were collected from each culture using a cork borer (5-mm diameter) after 6 days of incubation and placed into 10 mL of sterile distilled water and vortexed. The number of spores was counted under microscope with a hemacytometer.

The hydrophobicity of hyphal surface of colonies was tested by dropping 15 μL of 0.5% aqueous aniline blue on fully grown (6 d) colonies of wild-type Th33, Δ*thga3*, and R*thga3,* and observing the disappearance of the water or dye over an 8-h period (Mukherjee and Kenerley 2010).

### Antagonism and mycoparasitism assays

A dual culture technique (Dennis and Webster, 1971) was used for assessing the antagonistic activities of wild-type Th33 and its mutants against *R. solani*. Five-millimeter discs of *Trichoderma* and *R. solani* from 3-day-old cultures were placed in a PDA plate (90-mm diameter) 50 mm apart. A control plate was maintained with *R. solani* alone and incubated at room temperature (25 °C). Growth rate and colony morphology were assessed daily for six days. The percentage of growth inhibition was calculated using the equation RI = 100 × (R_2_—R_1_)/R_2_ (Li et al. 2019), where RI is the percentage of reduction in mycelial growth, R_1_ is the mycelial growth of *R. solani* in dual plates, and R_2_ is the mycelial growth of *R. solani* in the control. Three replicates were prepared for each treatment.

The slide culture method was used to investigate mycoparasitism of *Trichoderma* against *R. solani*. A glass microscope slide covered with a thin layer of 0.8% water agar (WA) was inoculated with 5-mm diameter mycelial discs of *T. harzianum* and *R. solani*, 10 mm apart from each other, and cultured at 25 °C. The regions where the hyphae of the two strains met were periodically observed under a light microscope as described previously (Jiang et al. 2016).

### Chitinase assays

The chitinase activities of *Trichoderma* strains were tested on colloidal chitin agar and liquid induced medium. The composition of colloidal chitin agar was MgSO_4_·7H_2_O (3 g/L), (NH_4_)_2_SO_4_ (3 g/L), KH_2_PO_4_ (2 g/L), citric acid (1 g/L), Tween-80 (200 μL/L), agar (15 g/L), and 5% colloidal chitin (5.25 g/L), pH 4.7. Colloidal chitin was prepared by the method of Sakai et al. (1998). Wild-type Th33, Δ*thga3*, and R*thga3* were inoculated independently at the center of colloidal chitin agar plates and cultured for 5 days at 25 °C. The colony growth and culture medium color changes were observed; the chitinase activity was proportional to the increasingly dark color of the medium. The chitinase activities of the wild type Th33, Δ*thga3*, and R*thga3* in culture filtrates were determined as described elsewhere (Fernandes et al. 2013). The absorbance was measured at a wavelength of 585 nm, and one unit of enzyme (U) was defined as the amount of enzyme necessary to produce 1 mg of *N*-acetylglucosaminidase per gram dry weight hyphae in 1 h. The test was repeated three times.

### Quantitative real-time PCR for detection of hydrophobin gene expression

Total RNA extraction and cDNA synthesis were performed as earlier described (Song et al. 2017). Primers were designed by Prime Express Software v2.0, and are listed in Additional file 2: Table S2. Ubiquitin-conjugating enzyme (UCE) gene (KX686115) was used for internal standard. Quantitative real-time PCR was performed on an ViiA 7 Real time system (ABI) system using a QuantiFast SYBR Green PCR Kit (400) (Qiagen). Three parallel experiments were performed of each sample in a total volume of 16 μL. The instrument was programmed for 2 min at 95 °C, followed by 40 cycles of 10 s at 94 °C, 10 s at 60 °C, and 40 s at 72 °C.

### Statistical analysis

The statistical software SAS 8.0 (SAS Institute, Inc., Cary, NC, USA) was used for ANOVA. Normality assumptions of the measured variables were checked, and no data transformation was required. Duncan's multiple range tests were used to compare the means obtained after each experiment. A value of P < 0.05 was considered statistically significant. The software Origin 8 was used for drawing.

## Results

### Construction and validation of *thga3* deleted and complemented mutants

The Thga3 gene was cloned from wild-type Th33 genome, which contains six exons and five introns and encoding 355 amino acids (GenBank Acc. No. KY937956). There was a single copy of *thga3* in the wild-type Th33 genome (data not shown). The *thga3* single-deleted strain Δ*thga3* with resistance to hygromycin B and *thga3* complemented mutant R*thga3* with resistance to G418 were obtained (Fig. 1c, d). For the Δ*thga3* strain, the PCR analysis confirmed the existence of the *hyg* sequence and the absence of the *thga3* sequence. Southern hybridization showed a single copy of the *hyg* sequence in Δ*thga3*. For the R*thga3* strain, PCR analysis confirmed the existence of *thga3*, *hyg*, and *neo* sequences. Southern hybridization showed a single copy of the *thga3* and *neo* sequence in the R*thga3* (Fig. 1c, d). The primers used for PCR, RT-PCR, and the probes for southern hybridization are listed in Additional file 1: Table S1.

### Morphology, growth, and conidiation

**Fig. 2:**
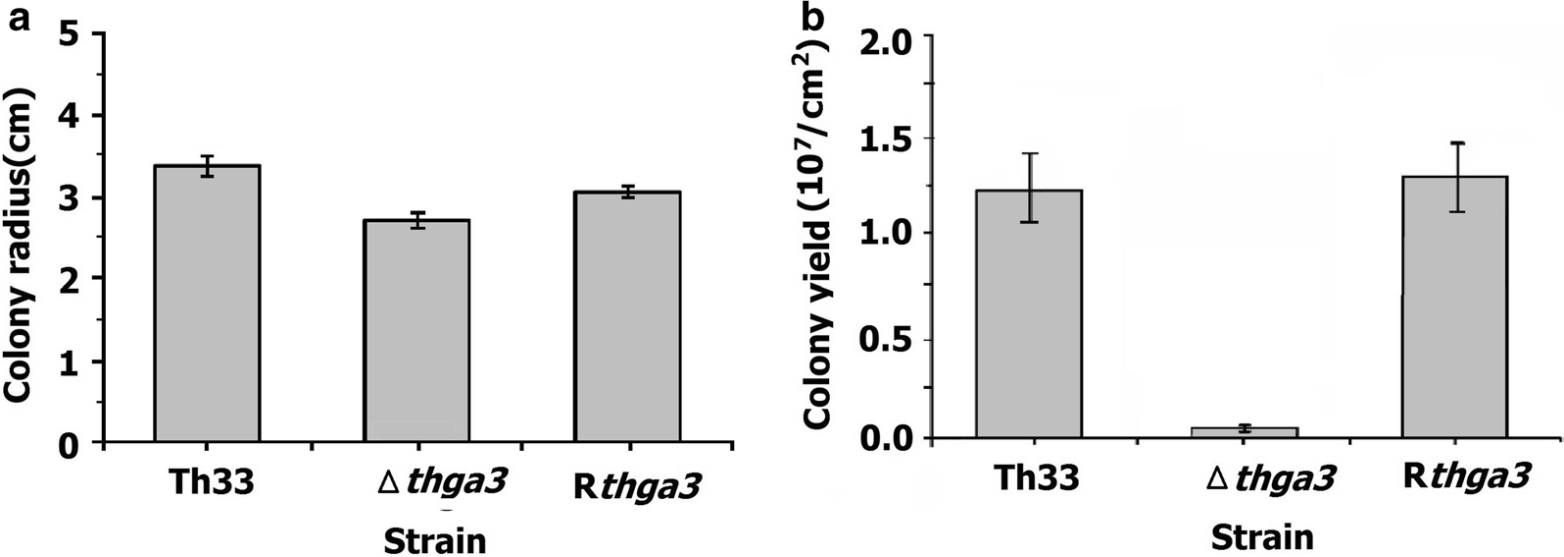
Growth and conidiation of the *T. harzianum* wild-type Th33, Δt*hga3*, and R*thga3*. **a** Growth of wild-type Th33, Δ*thga3*, and R*thga3* on PDA for 48 h at 25 °C. **b** Conidia yield of wild-type Th33, Δ*thga3*, and R*thga3*, on PDA for 6 d at 25 °C. Error bars represent the SD of three determinations for two independent experiments

When incubated on PDA medium, there were no significant differences in colony morphology, growth, and conidiation between wild-type Th33 and R*thga3*. Δ*Thga3* showed a 15% reduction (P < 0.001) in growth rate compared with wild-type Th33 (1.4 cm/day linear growth, compared with wild-type Th33 growth of 1.7 cm/day), when grown on PDA plates at 25 °C for 48 h (Fig. 2a), and the formation of aerial hyphae and conidia significantly decreased compared with wild-type Th33. The conidia yield of wild-type Th33, Δ*thga3*, and R*thga3* was 1.26 ± 0.17 × 10^7^ spores/cm^2^, 7.7 ± 0.58 × 10^5^ spores/cm^2^, and 1.32 ± 0.01 × 10^7^ spores/cm^2^, respectively, after culturing on PDA for 6 d. Δ*Thga3* conidia yield was approximately 94% lower (P < 0.001) than that of wild-type Th33 and R*thga3* (P < 0.0001) (Fig. 2b).

### Antagonistic activities against *R. solani*

**Fig. 3:**
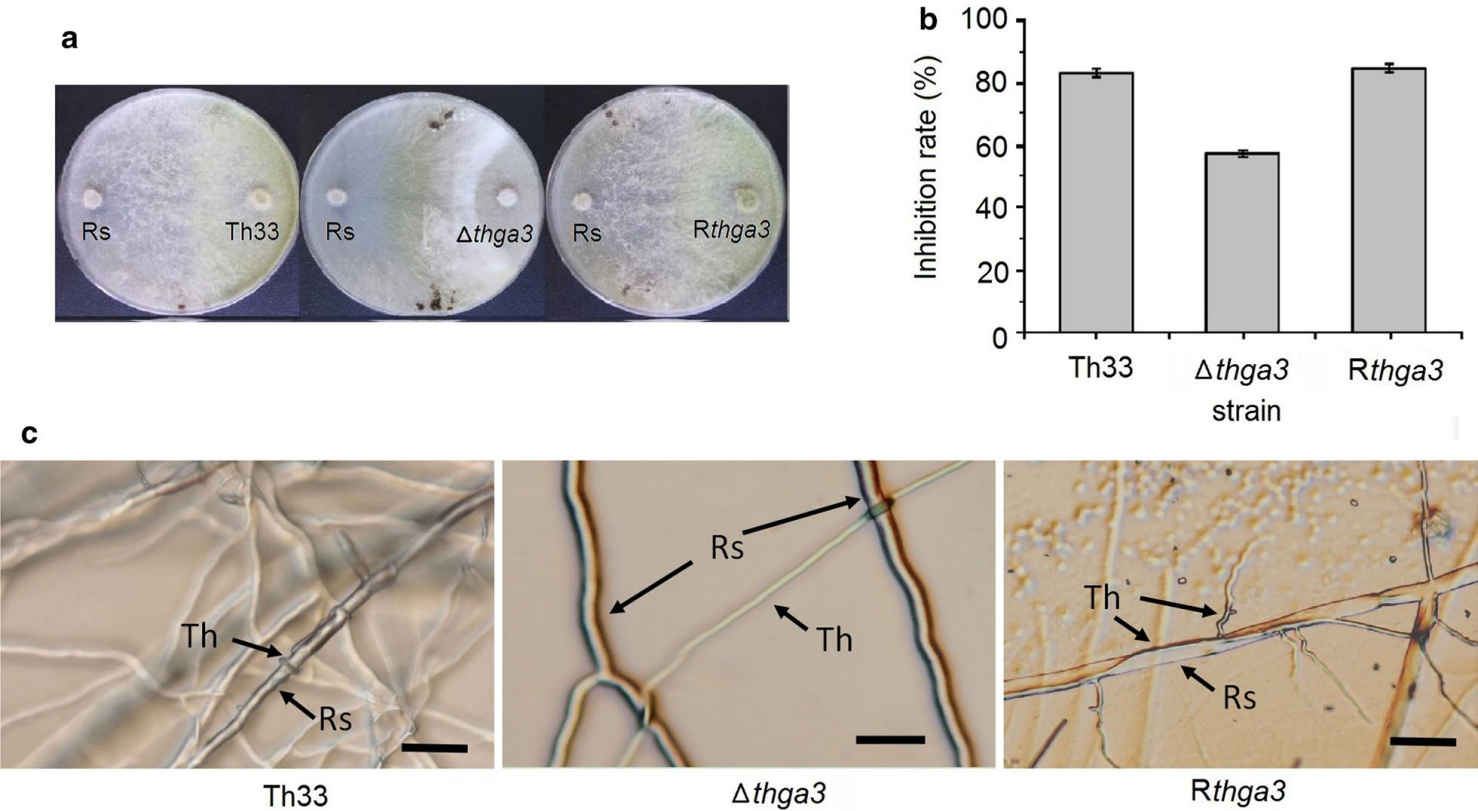
Antagonistic activities of the *T. harzianum* wild-type Th33, Δ*thga3*, and R*thga3* against *R. solani* (Rs). **a** Confrontation assay of wild-type Th33, Δ*thga3*, and R*thga3* against Rs. Cultures were grown on PDA plates for 10 d at 25 °C. **b** Inhibition ratio of the wild-type Th33, Δ*thga3*, and R*thga3* on the growth of Rs (grown on PDA plates for 6 d at 25 °C); error bars represent the SD of five determinations for two independent experiments. **c** Mycoparasitism of wild-type Th33, Δ*thga3*, and R*thga3* against Rs. For wild-type Th33 and R*thga3*, the hyphae grew along and coiled the hyphae of Rs; for Δ*thga3*, no coiled growth of the hyphae to the Rs was observed. Bars = 40 μm

When confront cultured with *R. solani*, all the wild-type Th33, Δ*thga3*, and R*thga3* could overgrow the colony of *R. solani* and further produce clusters of spores with different inhibitory effects (Fig. 3a). The growth inhibition ratios of wild-type Th33 and R*thga3* against *R. solani* were 83.83 ± 1.01% and 84.79 ± 0.90%, respectively, which were significantly higher than 58.24 ± 0.19% of Δ*thga3* after confront culturing for 6 d (P < 0.0001). The inhibition rate of Δ*thga3* decreased by about 31% compared to those of wild-type Th33 and R*thga3*. The hyphae of the wild-type Th33 and R*thga3* grew around the hyphae of *R. solani* (Fig. 3a, c), and part of the hyphae of *R. solani* underwent fragmentation. In Δ*thga3,* the hyphae of Δ*thga3* and *R. solani* grew independently and were not affected by each other, even when the hyphae of Δ*thga3* and *R. solani* came into contact, showing that Δ*thga3* lost its mycoparasitism ability against *R. solani* (Fig. 3b).

### Thga3 regulates the hyphal hydrophobicity and the expression of the hydrophobin gene

**Fig. 4:**
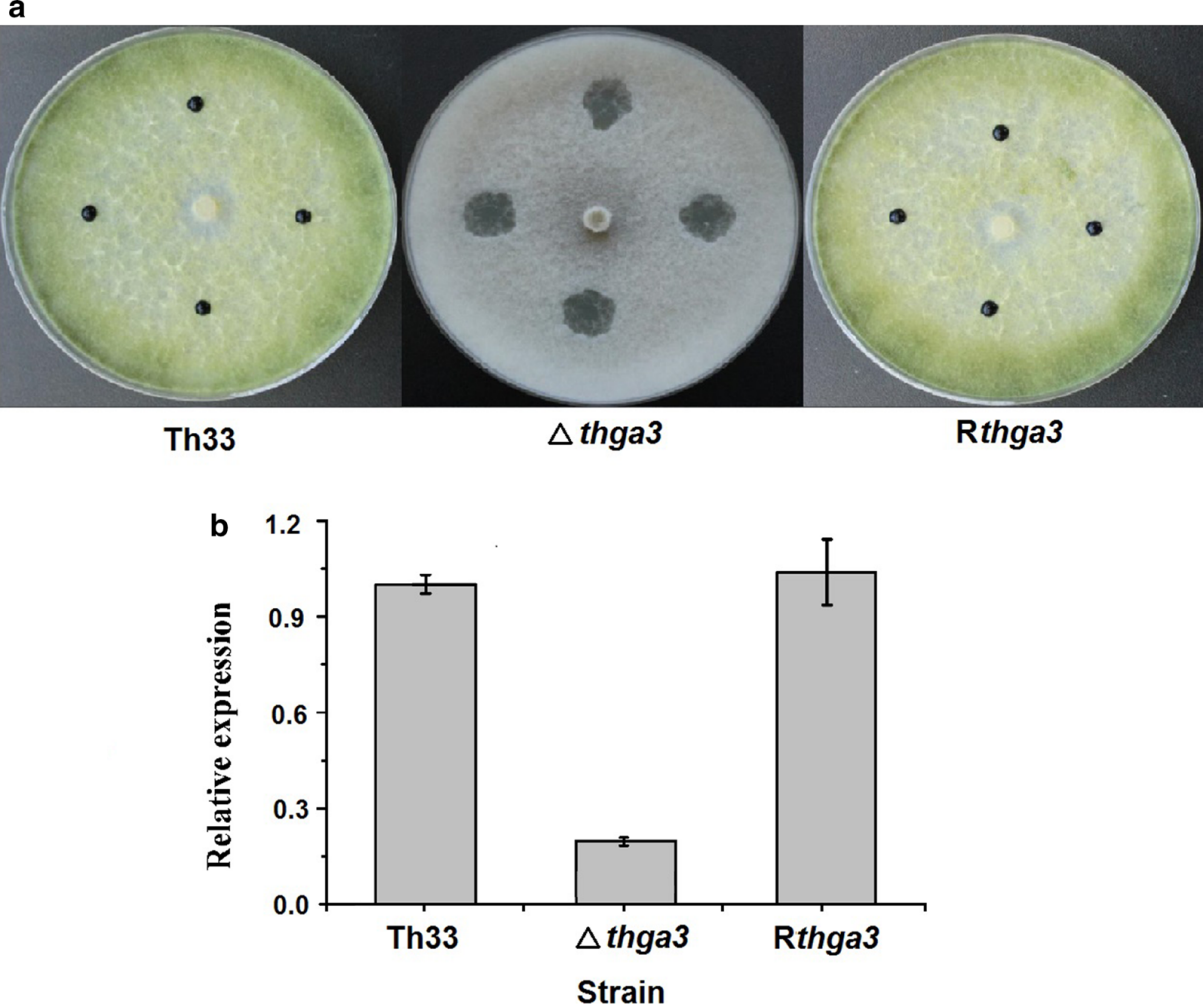
Hydrophobicity and expression of hydrophobin gene Tha_09745 of wild-type *T. harzianum* Th33, Δ*thga3*, and R*thga3*. **a** Hydrophobicity of wild-type *T. harzianum* Th33, Δ*thga3*, and R*thga3*. Fifteen microliters of 0.5% aqueous aniline blue was spotted onto colonies and imaged after 8 h. **b** Relative expression levels of Tha_09745 in wild-type Th33, Δ*thga3*, and R*thga3* grown on PDA plates for 7 days at 25 °C (fold-changes in mRNA expression relative to that of reference gene UCE). Error bars represent the SD of three determinations from two independent experiments

Wild-type Th33 and R*thga3* were highly hydrophobic, but Δ*thga3* was hydrophilic (Fig. 4a). The transcript level of the hydrophobin gene (tha_09745) of Δ*thga3* decreased by 80% compared with that of wild-type Th33 and R*thga3* as detected by qRT-PCR (Fig. 4b), whereas no significant differential expression of the other five hydrophobin genes was observed in Δ*thga3* compared with wild-type Th33 (Additional file 3: Fig. S1). The results showed that both the hydrophobicity and expression of Tha_09745 were positively regulated by Thga3, and we deduced that the expression level of Tha_09745 is correlated to the hyphal hydrophobicity of Th33.

### Thga3 regulates chitinase activities

**Fig. 5:**
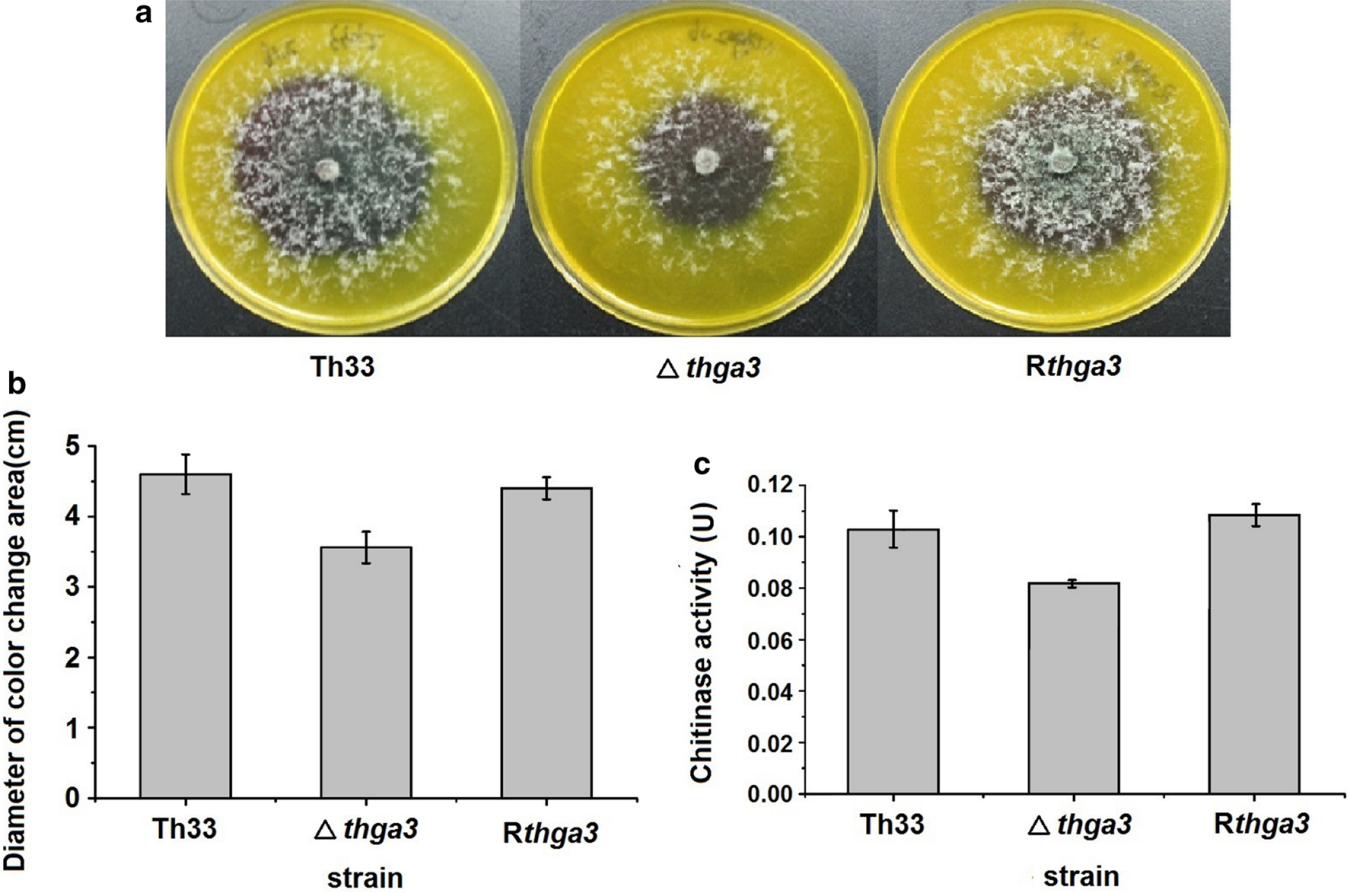
Chitinase activities of wild-type Th33, Δ*thga3*, and R*thga3*. **a** Discoloration of chitinase-inducing medium cultured with wild-type Th33, Δ*thga3*, and R*thga3* at 28 °C 5 days later. **b** Diameter of discolored area of chitinase-inducing medium cultured with wild-type Th33, Δ*thga3*, and R*thga3* at 28 °C 5 days later. **c** Chitinase activities of the culture filtrates of wild-type Th33, Δ*thga3*, and R*thga3.* Error bars represent the SD of three determinations from two independent experiments

When wild-type Th33, Δ*thga3*, and R*thga3* were growing on chitinase-inducing medium, the medium turned to dark purple with different discoloration ranges 5 days later (Fig. 5a). The diameters of purple areas of wild-type Th33 (4.6 ± 0.3 cm) and R*thga3* (4.4 ± 0.2 cm) were significantly greater than that of Δ*thga3* (3.6 ± 0.2 cm) (Fig. 5b). The corresponding activities of Δ*thga3* (82 ± 1.3 ug/h/mL) reduced by 23% compared with that of wild-type Th33 (100 ± 6.5 ug/h/mL) and R*thga3* (110 ± 5.1 ug/h/mL), showing that Thga3 positively regulated chitinase activity of Th33.

## Discussion

Gα3 subunits in various fungi possess multiple different regulatory functions. Thga3 in this study positively regulates the hyphal growth, which is similar with that of PGA3 (Gα3) in *Penicillium camemberti* (Hu et al. 2013), Gvm3 (Gα3) in *Valsa mali* (Song et al. 2017), and GanB (Gα3) in *Aspergillus nidulans* (Chang et al. 2004). Furthermore, FGA3 (Gα3) in *Fusarium oxysporum* shows no influence on vegetative growth (Guo et al. 2016). Gα3 subunits in *T. viride*, *T. atroviride*, and *Rhodospirillum* sp. negatively regulate conidiation (Schmoll et al. 2009; Zeilinger et al. 2005), and GanB (Gα3) in *Aspergillus nidulans* positively regulates conidial germination (Chang et al. 2004). Thga3 in this study positively regulates conidiation in Th33 and does not show influence on the conidial germination (data not shown).

GNA3 (Gα3) from *T. reesei* participates in cellulase activity and antimicrobial peptide synthesis (do Nascimento et al. 2009). Tga3 (Gα3) in *T. viride* and GzGPA2 (Gα3) in *F. graminearum* affect chitinase gene expression (Susanne et al. 2005), and PGA3 in *P. decumbens* regulates amylase and cellulase synthesis (Hu et al. 2013). In this study, the *thga3* knockout mutant shows a lower chitinase activity than those of the wild-type Th33 and *thga3*-complemented mutant R*thga3*, meaning that it positively regulates the chitinase activity of Th33, which is similar to Tga3 (Gα3) in *T. viride* and GzGPA2 (Gα3) in *F. graminearum*, but Thga3 shows no significant effect on cellulase and amylase activities (data not shown).

The regulatory functions of Gα3 in fungi are apparently affected by light. For example, Tga3 in *T. atroviride* shows hyper-conidiation in the dark with loss of *tga3* (Susanne et al. 2005), and GNA3 in *T. reesei* itself is induced by light exposure and regulates the expression of cellulase that is in response to light (Schmoll et al. 2009). However, the regulatory functions of Thga3 on the growth, conidiation, and chitinase activity of Th33 in this study seem to have no correlation with light (data not shown).

Based on aforementioned comparison and analysis, we speculated that the role of Ga3 is diverse and complicated in different fungi species, which could influence growth, sporulation, chitinase and cellulase activities, as well as their responses to light in different strains. However, the reports concerning the function of Ga3 in fungi are still limited, and its regulation mechanism is not revealed yet. Therefore, study on the regulatory mechanisms of Ga3 will help us to uncover the difference of its function in different strains”.

*T. harzianum* Th33 in this study is a biocontrol fungi used in controlling plant fungal diseases caused by *R. solani*, *Sclerotium rolfsii*, *Sclerotinia sclerotiorum*, *F. oxysporum*, and *Pythium* spp. (Sun et al. 2016). Deleting *thga3* resulted in a significant decrease in antagonistic and mycoparasitic ability, which is similar to GzGPA2 in *F. graminearum*, wherein deletion of *GzGPA2* caused reduced pathogenicity and increased chitin accumulation in the cell wall (Yu et al. 2008). Chitinase was reported to have antifungal activities (Lorito et al. 1993). We thus deduced that the reduced chitinase activities of Δ*thga3* reduces the antagonistic and mycoparasitic ability of *Trichoderma* against *R. solani*.

Hydrophobins are small cysteine-rich surface active proteins produced by fungi on the outer surface of cell walls. The roles of hydrophobins in surface hydrophobicity, conidiation, fruit body formation, recognition, and adhesion onto the host surface and virulence have been investigated (Dubey et al. 2014; Minenko et al. 2014; Zhang et al. 2011). In this study, wild-type Th33 and R*thga3* are hydrophobic, whereas the hyphae of Δ*thga3* is hydrophilic. The Th33 genome (GenBank Acc. No. PRJNA272949) harbors six hydrophobin-encoding genes. We detected the expressions of all of the six hydrophobin genes in wild-type Th33, Δ*thga3*, and Rthga3 (data not shown). Only the expression of Tha_09745 significantly decreased in Δ*thga3* compared with wild-type Th33 and R*thga3*, and there was no significant difference between wild-type Th33 and R*thga3*, which corresponded with the hydrophobicity phenotype of the strains. We deduced that the expression level of Tha_09745 is correlated to the hyphal hydrophobicity of Th33, and loss of hydrophobicity may affect the surface recognition and mycoparasitism of Th33 against *R. solani,* although this requires further investigation.

Both G proteins and hydrophobin in fungi have a variety of regulatory functions, and numerous studies on this property have been conducted (Huang et al. 2015; Espino-rammer et al. 2013; Khalesi et al. 2015). However, studies on the correlation between the G proteins and hydrophobin in fungi are limited, Segers and Nuss (2003) reported that Gα negatively regulated hydrophobin gene expression in the chestnut blight fungus *Cryphonectria parasitica*. In this study, we found for the first time that Gα in *Trichoderma* positively regulates the expression of the hydrophobin gene and hyphal hydrophobicity, which is also correlated to the growth, conidiation, and mycoparasitism of Th33. Our findings may be used in future studies on the regulatory mechanism of the G signal system in fungi.

## Supplementary Information

**Additional file 1: Table S1.** Primers used for deletion and complementation of Thga3.

**Additional file 2: Table S2.** Primers for qRT-PCR detection the expression of six hydrophobin genes and UCE (ubiquitin-conjugating enzyme) gene as reference gene in wild-type Th33 and *thga3* deletion strain Δ*thga3*.

**Additional file 3: Figure S1.** The relative expression of the five hydrophobin genes in wild-type Th33 and Δ*thga3*.

## Data Availability

Not applicable.
