# Supplementary material for: Gα3 subunit Thga3 positively regulates conidiation, mycoparasitism, chitinase activity, and hydrophobicity of Trichoderma harzianum
Source: AMB Express. 2020 Dec 17;10:221. doi: 10.1186/s13568-020-01162-9 (PMC7746536; doi:10.1186/s13568-020-01162-9)
Supplement: Supplementary file 1 — Additional file 1: Table S1. Primers used for deletion and complementation of Thga3. [file 13568_2020_1162_MOESM1_ESM.doc]

**Table S1 Primers for deletion and complementation of Thga3 gene**

| Primers | Sequences (5' to 3') | Functions |
| --- | --- | --- |
| thga3-5up | cgctctagaactagtaaacgcaccacaatgaatg | Amplified the 5’ homologous sequence to construct the *Δthga3* strain, length 1110 bp |
| thga3-5do | tcagttatcgaattccgggatgcttcagatagagtc |
| thga3-3up | ccagt tatcaagcttgcatttttctcccataacg | Amplified the 3’ homologous sequence to construct the Δ*thga3* strain, length 1036 bp |
| thga3-3do | gtcctcagcctcgaggtattcatccatcaaaaaggc |
| thga3-up | atggcaaacccaccttttg | Amplified thga3 sequence for cloning, PCR and RT-PCR indentification, length 1562bp and 1133bp |
| thga3-do | ttgaccgccgcaaaaacga |
| hyg-up | cacgataacttgtgcgtttgtc | Amplified *hyg* sequence to construct and confirmation of the Δ*thga3* strain, length 1221 bp |
| hyg-do | ggtcggcatctactctattcct |
| Pthga3-up | cgattcgtcaagtgcttagag | Amplified *thga3* sequence，Probe 1 for Southern hybridization, length 371 bp |
| Pthga3-do | tcggtcggtagttgtagagc |
| Phyg-up | tccaacaatgtcctgacg | Amplified *hyg* sequence, Probe 2 for Southern hybridization, length 426 bp |
| Phyg-do | tctattcctttgccctcg |
| PG418-up | ttgaacaagatggattgcacgcagg | Amplified G418 sequence, Probe3 for Southern hybridization, length 425 bp |
| PG481-do | tcgctcgatgcgatgtttcc |
| G418-up | tgcctcagcgaattcactcactatagggcgaattgg | Amplified G418 sequence, to construct the R*thga3* strain, length 1258 bp |
| G418-do | ggtatcgataagcttggagcctgaatgttgagtggaatg |
| PgpdA-up | cgccctatagtgaggaattcccttgtatctctacac | Amplified PgpdA gene sequence to construct the R*thga3* strain, length 2130 bp |
| PgpdA-do | ccaaaaggtgggtttgccattgggaaaagaaagagaaaag |
| thga3-up1 | tttctctttcttttcccaatggcaaacccaccttttgg | Amplified *thga3* sequence, to construct the R*thga3* strain, length 1598 bp |
| thga3-do1 | gtaacgttaa gtggatcctt agaggatacc cgagtcc |
| TtrpC-up | gactcgggtatcctctaaggatccacttaacgttactg | Amplified TtrpC sequence to construct the R*thga3* strain, length 770 bp |
| TrpC-do | cgctctagaactagttcgagtggagatgtggag |
