# Supplementary material for: Gα3 subunit Thga3 positively regulates conidiation, mycoparasitism, chitinase activity, and hydrophobicity of Trichoderma harzianum
Source: AMB Express. 2020 Dec 17;10:221. doi: 10.1186/s13568-020-01162-9 (PMC7746536; doi:10.1186/s13568-020-01162-9)
Supplement: Supplementary file 2 — Additional file 2: Table S2. Primers for qRT-PCR detection the expression of six hydrophobin genes and UCE (ubiquitin-conjugating enzyme) gene as reference gene in wild-type Th33 and thga3 deletion strain Δthga3. [file 13568_2020_1162_MOESM2_ESM.doc]

**Table S2 Primers for qRT-PCR of six hydrophobin genes**

| Primers | Sequences (5' to 3') | Functions |
| --- | --- | --- |
| 773-up | aacagcaacaagatgggc | Amplified Tha_00773 fragment，length 124bp |
| 773-do | ttacctgagtgttcgtgcc |
| 2696-up | ttgccctgctgttgtttc | Amplified Tha_02696 fragment，length 104bp |
| 2696-do | tcctgtctttaggctccttg |
| 3110-up | ggatgcccaggtggtattac | Amplified Tha_03110 fragment，length 119 bp |
| 3110-do | tggaagatgccgacattg |
| 4012-up | caaccctctgtgctgtgatac | Amplified Tha_04012 fragment，length 101 bp |
| 4012-do | agacgctgccaaaggact |
| 4345-up | caccaatactcaggtaaatgcc | Amplified Tha_04345 fragment，length 116 bp |
| 4345-do | agcaaatgatggtctcttcg |
| 9745-up | tcacttctcccctcaacccc | Amplified hydrophobin Tha_09745 fragment，length 122 bp |
| 9745-do | atcctggcacaaaacaccga |
| UCE-up | gtggcggcagcacttgttat | Amplified UCE gene fragment as internal standard, length 125 bp |
| UCE-do | atgacgaacgaaaagcaccg |
