# Supplementary figures and images for: Gα3 subunit Thga3 positively regulates conidiation, mycoparasitism, chitinase activity, and hydrophobicity of Trichoderma harzianum
Source: AMB Express. 2020 Dec 17;10:221. doi: 10.1186/s13568-020-01162-9 (PMC7746536; doi:10.1186/s13568-020-01162-9)

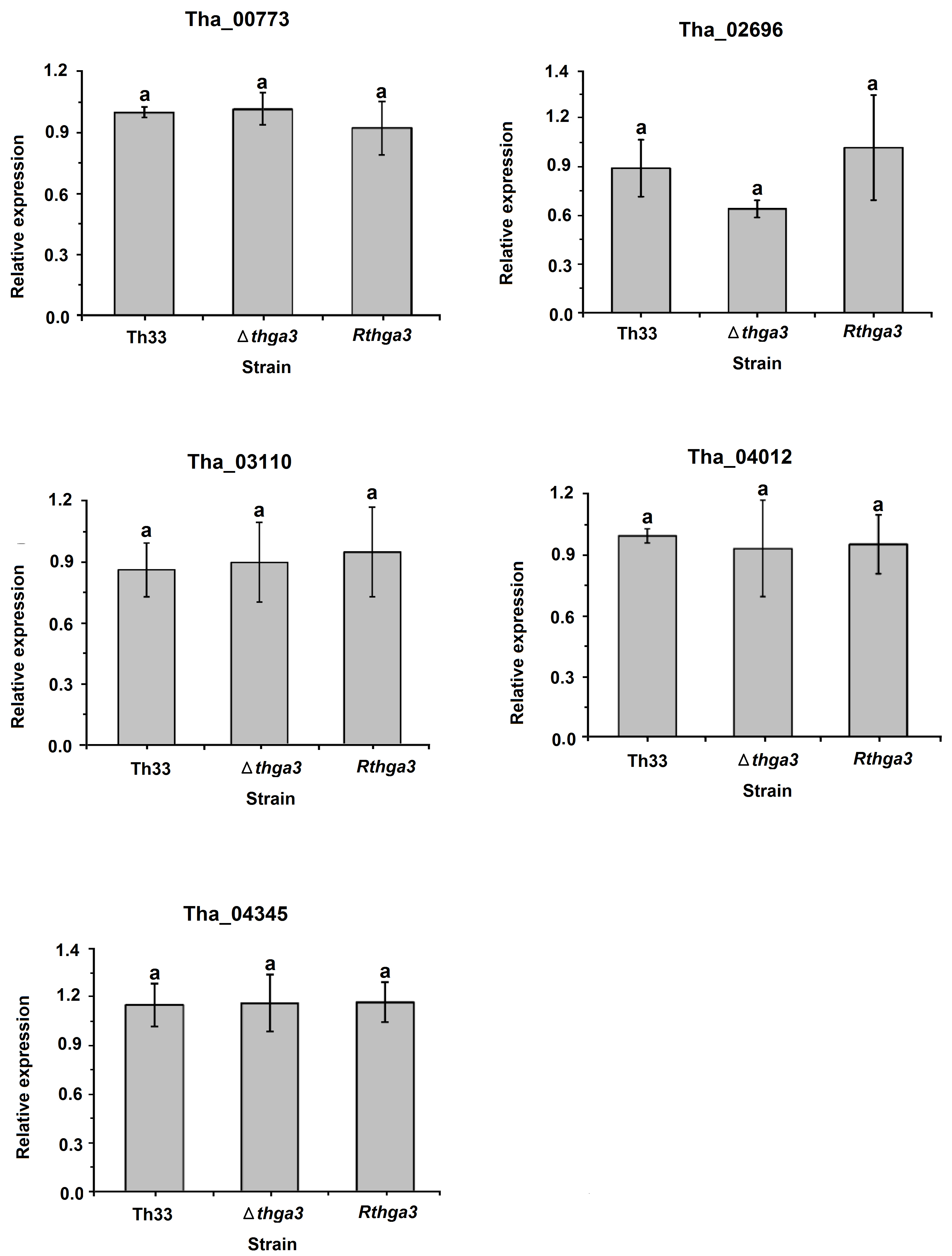

Supplement: Supplementary file 3 — Additional file 3: Figure S1. The relative expression of the five hydrophobin genes in wild-type Th33 and Δthga3. [file 13568_2020_1162_MOESM3_ESM.jpg]
